# Archimedes Optimization Algorithm-Based Feature Selection with Hybrid Deep-Learning-Based Churn Prediction in Telecom Industries

**DOI:** 10.3390/biomimetics9010001

**Published:** 2023-12-19

**Authors:** Hanan Abdullah Mengash, Nuha Alruwais, Fadoua Kouki, Chinu Singla, Elmouez Samir Abd Elhameed, Ahmed Mahmud

**Affiliations:** 1 Department of Information Systems, College of Computer and Information Sciences, Princess Nourah bint Abdulrahman University, P.O. Box 84428, Riyadh 11671, Saudi Arabia; 2 Department of Computer Science and Engineering, College of Applied Studies and Community Services, King Saud University, P.O. Box 22459, Riyadh 11495, Saudi Arabia; 3 Department of Financial and Banking Sciences, Applied College at Muhail Aseer, King Khalid University, Abha 61413, Saudi Arabia; falkoki@kku.edu.sa; 4 Department of Computer Science, University of the People, Pasadena, CA 91101, USA; 5 Department of Computer Science, College of Post-Graduated Studies, Sudan University of Science and Technology, Khartoum 11111, Sudan; 6 Research Center, Future University in Egypt, New Cairo 11835, Egypt

**Keywords:** bio-inspired algorithms, telecom industry, feature selection, metaheuristics, churn prediction

## Abstract

Customer churn prediction (CCP) implies the deployment of data analytics and machine learning (ML) tools to forecast the churning customers, i.e., probable customers who may remove their subscriptions, thus allowing the companies to apply targeted customer retention approaches and reduce the customer attrition rate. This predictive methodology improves active customer management and provides enriched satisfaction to the customers and also continuous business profits. By recognizing and prioritizing the relevant features, such as usage patterns and customer collaborations, and also by leveraging the capability of deep learning (DL) algorithms, the telecom companies can develop highly robust predictive models that can efficiently anticipate and mitigate customer churn by boosting retention approaches. In this background, the current study presents the Archimedes optimization algorithm-based feature selection with a hybrid deep-learning-based churn prediction (AOAFS-HDLCP) technique for telecom companies. In order to mitigate high-dimensionality problems, the AOAFS-HDLCP technique involves the AOAFS approach to optimally choose a set of features. In addition to this, the convolutional neural network with autoencoder (CNN-AE) model is also involved for the churn prediction process. Finally, the thermal equilibrium optimization (TEO) technique is employed for hyperparameter selection of the CNN-AE algorithm, which, in turn, helps in achieving improved classification performance. A widespread experimental analysis was conducted to illustrate the enhanced performance of the AOAFS-HDLCP algorithm. The experimental outcomes portray the high efficiency of the AOAFS-HDLCP approach over other techniques, with a maximum accuracy of 94.65%.

## 1. Introduction

Telecommunications has become one of the most large-scale industries in developed countries. The technological developments and a large number of operators increase the range of challenges encountered by the industry [1]. Companies are actively working to survive in this competitive market, for which several approaches are being followed [2]. In order to generate high revenues, three key policies are followed, such as gaining new customers, promoting the existing customers, and raising the retention time of the customers. Comparing these policies and taking the return on investment (RoI) cost of all into account, it can be inferred that the third policy is the most profitable approach [3], since retaining a present customer costs considerably less than gaining a new one. Further, it is also regarded as a simple task compared to the upselling plan. In order to implement the third policy, companies need to reduce the ability of customer churn [4]. Alternatively, the prediction of the customers who are likely to leave the network can help in retaining the customer and, thus, indicates a possibly massive increase in profit if it is implemented in the early phase [5]. Various studies have established that the machine learning (ML) technique is extremely effective in predicting the churning customers. This approach is implemented based on the knowledge gained from prior data [6].

Big data tasks can be performed easily with the help of artificial intelligence (AI) technology without much effort from the sales and customer support teams [7]. So, it is crucial to incorporate the AI in financial activities that contain social marketing, sales, customer relationship management (CRM), and so on to effectively attract the customers and gain their trust. Since AI is a significant part of social networks and other electronic marketing sites, it is crucial to understand how to utilize, change, and execute these sites in an efficient manner [8]. Customer behavior analysis seriously affects the social networking and other marketing actions of the company by permitting highly customized and predictive marketing activities. By analyzing the customer data, the companies increase their vision on what can resonate with their viewers [9]. Businesses employ such data to engage in highly efficient social media and marketing activities. It can successively result in greater customer support and conversion rates. Also, the deep learning (DL) techniques can support companies in terms of optimization and automation of their promotional activities, thus saving resources and time, while it also enhances the firm’s overall effectiveness. Recently, metaheuristic algorithms [10] have been widely used for hyperparameter tuning of the DL models. A few such metaheuristics include monarch butterfly optimization (MBO) [11], slime mold algorithm (SMA) [12], moth search algorithm (MSA) [13], hunger games search (HGS) [14], Runge Kutta method (RUN) [15], colony predation algorithm (CPA) [16], weighted mean of vectors (INFO) [17], Harris hawks optimization (HHO) [18], rime optimization algorithm (RIME) [19], etc.

In this background, the current study introduces the Archimedes optimization algorithm-based feature selection with hybrid deep-learning-based churn prediction (AOAFS-HDLCP) technique for telecom companies. The objective of the proposed AOAFS-HDLCP method is to predict the churning customers so as to increase the customer retention activities in the telecom industry. In the presented AOAFS-HDLCP technique, the AOAFS approach is intended to choose an optimal set of features. It has the following benefits, i.e., fast convergence rate and a fine balance between local and global search capacity, while resolving continuing problems. The current study involves the convolutional neural network with an autoencoder (CNN-AE) model for churn prediction. Further, the thermal equilibrium optimization (TEO) technique has been applied to the hyperparameter tuning method to boost the outcomes of the CNN-AE model. An extensive experimental analysis was conducted to illustrate the enhanced performance of the AOAFS-HDLCP method. Briefly, the major contributions of this research are given below:

- An intelligent AOAFS-HDLCP method including AOAFS, CNN-AE classification, and TEO-based hyperparameter tuning is introduced for churn prediction. The AOAFS-HDLCP method does not exist in the literature to the best of the authors’ knowledge.
- The AOAFS method is designed to detect the essential attributes from the telecom industry’s complex datasets, thus enhancing the efficiency and effectiveness of the churn prediction process.
- The CNN-AE model is employed for the churn prediction process, which represents a significant contribution to the research community. It can capture intricate patterns and relationships in the data, thus potentially improving the accuracy of churn prediction compared with the rest of the traditional approaches.
- A TEO technique has been developed to fine-tune the model parameters of the CNN-AE model in an effective manner so as to optimize the performance in terms of predicting customer churn.

## 2. Related Works

The authors in the literature [20] introduced the AI with Jaya optimization algorithm (JOA)-based churn prediction for data exploration (AIJOA-CPDE) method. In this algorithm, a primary step of feature selection was introduced by employing the JOA approach for the selection of feature sets. The proposed system utilized a bidirectional LSTM (BLSTM) algorithm for churn prediction. Finally, the chicken swarm optimization (CSO) method was applied in this study for hyper-parameter optimization. Kozak et al. [21] considered customer churn management to validate the efficiency of swarm intelligence machine learning (SIML) techniques. The aims of this study were of two-fold: for the existence of particular features and the objective in customer churn management and validating whether the adapted SIML technique increased the efficiency of churn-related segmentation and decision-making method. Saha et al. [22] studied ensemble learning approaches, namely, xgboost (XGB), bagging and stacking, Adaboost, gradient boosting (GBM), extremely randomized tree (ERT), and random forest (RF), standard classification algorithms, such as LR, ANN, DT, and KNN, and the DL-CNN approach in order to select the best method for developing the CCP technique.

In the literature [23], the authors developed the dynamic customer churn prediction (CCP) method for business intelligence by applying text analytics with a metaheuristic optimizer (CCPBI-TAMO) method. Additionally, the LSTM with stacked AE (LSTM-SAE) algorithm was also implemented for the classification of the feature-minimized data. Faritha Banu et al. [24] suggested the AI-based CCP for Telecommunication Business Markets (AICCP-TBM) method in which the chaotic SSO-based FS (CSSO-FS) algorithm was utilized for selecting the superior feature set. Additionally, the fuzzy-rule-based classifier (FRC) was exploited for differentiating the non-churn customers and churners. The quantum behaved particle swarm optimization (QPSO) approach was applied in this study to select the membership roles for the FRC algorithm.

In the study conducted earlier [25], the stacked bidirectional LSTM (SBLSTM) and RNN models were developed for AOA from CCP. The aim of the presented approach was to forecast the existence of customer churn from the insurance company. Primarily, the AOA approach conducted the preprocessing of the data to change the new data into a valuable format. Moreover, the SBLSTM-RNN algorithm was utilized in this study for distinguishing the churn and non-churn customers. In the literature [26], the authors created an ML approach that can forecast the effective churn for the telecom companies. The outcomes can be used in an appropriate manner, i.e., use marketing retention approaches to retain the customers as and when time passes. In this method, the authors employed recent databases and made use of preprocessing systems such as bivariate and univariate analyses and employed data visualization methods to understand the database correctly. Alshamari [27] intended to analyze and measure the user approval for the services rendered by the Saudi Telecom Company (STC), Mobily, and Zain. This kind of SA has been a dominant parameter and has been utilized to create a significant business decision in enhancing the satisfaction as well as the loyalty of the customers. In this case, the author established new approaches based on DL technique for analyzing the percentage of customer satisfaction using the openly accessible database, i.e., AraCust.

The existing literature on CCP has made significant strides in leveraging both ML and DL techniques to identify the potential churners. However, a notable research gap persists in adequately addressing the critical aspects of feature selection and hyperparameter tuning within this context. Though comprehensive studies have been conducted earlier on individual aspects of CCP, the simultaneous consideration of feature selection and hyperparameter tuning remains an underexplored territory. Feature selection plays an important role in improving the efficacy of the model by detecting the most informative variables, thus reducing both noise and computation. At the same time, hyperparameter tuning is crucial for fine-tuning the model’s performance and generalization. The synergy between these two crucial aspects can potentially yield highly efficient and accurate churn prediction methods. However, the existing research often overlooks this synergy, thus resulting in suboptimal predictive abilities. Bridging this research gap is a vital element to unlock the maximum potential of CCP algorithms. This can further offer the businesses highly efficient mechanisms for customer retention and improved decision-making processes in extremely competitive industries.

## 3. The Proposed Model

**Figure 1:**
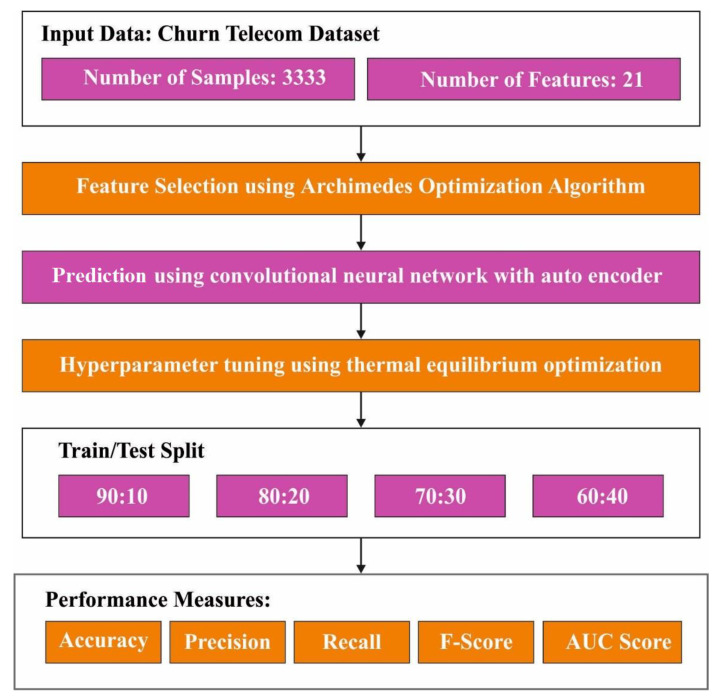
Overall procedure of the AOAFS-HDLCP system.

In this article, the AOAFS-HDLCP system has been proposed for churn prediction in the telecom industry. The objective of the AOAFS-HDLCP method is to obtain churn prediction so as to increase the customer retention in the telecom industry. In the presented AOAFS-HDLCP technique, the AOAFS approach, CNN-AE classification, and TEO-based hyperparameter tuning are introduced. Figure 1 exhibits the working procedure of the AOAFS-HDLCP approach.

### 3.1. Stage I: Feature Selection Using AOA

In this study, the AOA is designed to choose the optimum feature set. The fundamental condition of AOA is based on Archimedes’ physical law of buoyancy [28]. AOA is an effective model for the optimization process since it can balance the tradeoff between exploration and exploitation phases, thus making it suitable for managing difficult and multidimensional search spaces. Inspired by the Archimedes’ principle of buoyancy, the AOA method formulates an effective way for its searching mechanism based on the fitness landscape, thus enabling effective convergence towards the optimal solution. It is highly adaptable, integrated to the ability of escaping the local minima and well suited for addressing real-world problems across various domains. Since the feature selection process identifies highly relevant features, the AOA’s adaptability and capacity to discern informative features from a multitude of possibilities prove to be invaluable. With dynamic adjustment of the searching process based on the dataset characteristics, the AOA performs well in the detection of optimum feature subsets. It results in improved model interpretability, reduced computational complexity, and improved generalization performance.

AOA is a new metaheuristic algorithm, derived from the Archimedes’ principle. Similar to other population-based metaheuristic techniques, the AOA technique begins its search method with an initial population and a random volume, density, and acceleration. Following is the list of steps followed in AOA method. 

Step 1. Initialize the population location, volume, density, and acceleration using the following Equation (1):

(1) $$X_{i}=lb_{i}+rand\times \left(ub_{i}-1b_{i}\right);i=1,2,\ldots ,N,\;acc_{i}=lb_{i}+rand\times \left(ub_{i}-1b_{i}\right);i=1,2,\ldots ,N,\;den_{i}=rand\;\left(N,D\right)\;vol_{i}=rand\;\left(N,D\right)$$

where the population number and dimension of the search range are N and D, respectively. The $i^{th}$ object in the N population is $X_{i}$. The lower and upper limitations of the search range are $lb_{i}$ and $ub_{i}$, respectively. N×D dimensional matrix that can be calculated randomly by the system function is denoted by  rand(N,D). Volume, density, and acceleration of the $i^{th}$ object are $vol_{i},\;den_{j},$ and $acc_{i}$, correspondingly. Next, the individual $X_{best}$ with the optimum fitness value and the respective $acc_{best},\;den_{best}$, and $vol_{best}$ are chosen [29].

Step 2. Upgrade the density and volume of the $\left(t+1\right)th$ iteration of the $i^{th}$ objectas given below.

(2) $$den_{i}^{t+1}=den_{i}^{t}+rand\times \left(den_{best}-den_{i}^{t}\right),\;\;\;vol_{i}^{t+1}=vol_{i}^{t}+rand\times \left(vol_{best}-vol_{i}^{t}\right),$$

In Equation (2), the global optimum values of density and volume are denoted by $den_{best}$ and $vol_{best}$, correspondingly.

Step 3. Compute the density decline factor d and the parameter TF, which creates a balance between global and local convergence capability of the AOA method.

(3) $$TF=exp\left(\frac{t-t_{max}}{t_{max}}\right),$$

In Equation (3), the maximum and the existing iterations are denoted by $t_{max}$ and t, respectively. Here, TF rises with the iteration number, until TF = 1.

(4) $$d^{t+1}=exp\left(\frac{t_{max}-t}{t_{max}}\right)-\left(\frac{t}{t_{max}}\right),\;$$

In Equation (4), as the iteration number increases, d reduces and the search is transported to the bounded area that has been detected [30].

Step 4. When TF≤0.5, then the exploration and collision takes place between the objects. Using the following equation, the acceleration is updated.

(5) $$acc_{i}^{t+1}=\frac{den_{mr}+vol_{mr}\times acc_{mr}}{den_{i}^{t+1}+vol_{i}^{t+1}},\;\;mr=rand,$$

In Equation (5), acceleration, volume, and density of the $i^{th}$ individual at (t+1)th iteration are denoted by $c_{i}^{t+1},$ $vol_{i}^{t+1}$, and $\;den_{i}^{t+1}$, correspondingly. The $c_{i}^{t+1},$ $vol_{i}^{t+1}$, and $\;den_{i}^{t+1}$ of the random individuals are denoted by $acc_{mr},$ $den_{mr},$ $and\;vol_{mr}$, correspondingly.

When TF>0.5, the exploitation stage and no collision between the objects takes place. So, the acceleration is updated as given below.

(6) $$acc_{i}^{t+1}=\frac{den_{best}+vol_{best}\times acc_{best}}{den_{i}^{t+1}+vol_{i}^{t+1}},\;$$

Next, using the following equation, the acceleration is normalized.

(7) $$acc_{i,norm}^{t+1}=u\times \frac{acc_{i}^{t+1}-min\left(acc\right)}{max\left(acc\right)-min\left(acc\right)}+l,\;\;$$

In Equation (7), the range of normalization and fixed value at 0.9 and 0.1 are u and l, correspondingly. The step percentage of each agent change is $acc_{i,norm}^{t+1}$. When the object i is far from the global optima, then $acc_{i,norm}^{t+1}$ value would be higher, which implies that the object is in the exploration stage.

Step 5. When TF≤0.5, then the location of the population X is updated using the equation below.

(8) $$X_{i}^{t+1}=X_{i}^{t}+C_{1}\times rand\times acc_{i,norm}^{t+1}\times d\times \left(X_{rand}-X_{i}^{t}\right),\;$$

In Equation (8), $C_{1}$ is a constant equivalent to 2. Or else, when TF>0.5, the location of the population X is updated using Equation (9):

(9) $$X_{i}^{t+1}=X_{best}^{t}+F\times C_{2}\times rand\times acc_{i,norm}^{t+1}\times d\times \left(T\times X_{best}-X_{i}^{t}\right),$$

Here, $C_{1}$ is a constant equivalent to 6. $T=C_{3}\times TF$; T rises with time. The parameter F changes the movement’s direction and is evaluated by Equation (10):

(10) $$P=2\times rand-C_{4},\;F=\left\{\begin{array}{l} +1,\;\;ifP\leq 0.5,\\ -1,\;\;ifP>0.5, \end{array}\right.\;\;$$

where $C_{3}$ and $C_{4}$ balance the direction of the movements to adjust the capability of the model so as to escape the local optima.

Step 6. Evaluation. Based on the updated population, the individual with the optimal fitness and their acceleration, density, and volume are selected. The procedure is reiterated until the maximal iteration is obtained [31].

The FF of the AOA-FS technique considers the classification outcomes and the amount of features selected. It diminishes the set size of the selected features and increases the classification outcomes. Hence, the FF is used for evaluating the individual solutions:

(11) $$Fitness=\alpha \;*\;ErrorRate+\left(1-\alpha \right)\;*\;\frac{\#SF}{\#All\_F},\;$$

In Equation (11), ErrorRate implies the classifier error rate based on the selected features. ErrorRate is estimated as a percentage of incorrect classification to the amount of classifications made in the range of [0,1]. #SF shows the number of features selected and #All_F denotes the total quantity of features in the original dataset. α controls the prominence of classification quality and the subset length. α is fixed as 0.9 in the current study. 

### 3.2. Stage II: Churn Prediction Using CNN-AE Model

**Figure 2:**
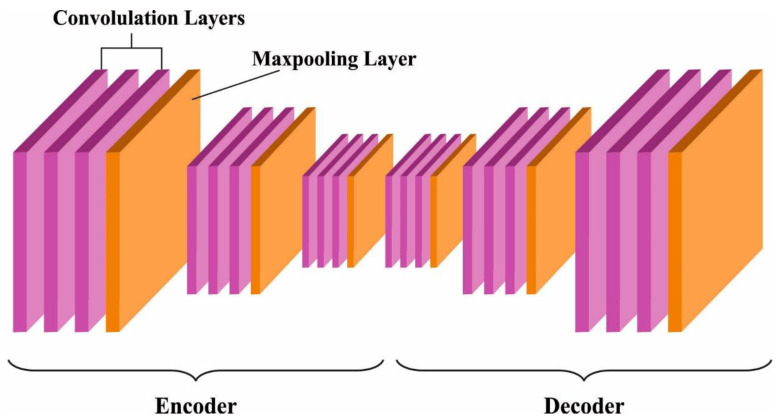
Structure of CNN-AE.

The CNN-AE model is used for churn prediction. CNN model is a kind of DL method and is one of the state-of-art techniques for CV applications, owing to its considerable benefits [32]. CNN technique has a primary benefit, i.e., feature learning, and it can extract and learn relevant features. Due to its deep architecture, the CNN technique also learns from abundant datasets. Feature extraction is a main and challenging problem for pattern prediction. The features are highly essential since they represent the image properties. CNN is a DL approach used for the extraction of features that give a self-learning layer. The component in the encoded vector does not mean to encode a single feature. In the decoding network, masses of parameters exist while a combination could encode and construct a vast number of features. Thus, the CNN-AE technique is used to implement the unsupervised learning for dimension reduction and feature extraction. The distance between the vectors is much more rapid to compute since the smaller feature is projected to be a low dimension. Figure 2 demonstrates the infrastructure of the CNN-AE model.

CAE has a similar structure to CNN that comprises pooling layers and convolutional filters. However, the only difference between CNN and CAE is that both input and output nodes have equal dimensions in CAE. The recreated data are compared to the input dataset. The learning method is not reliant on the labeled dataset. The CNN-AE is a category of unsupervised learning method, while CNN is a kind of DL method with multiple convolutional layers. It is primarily exploited for feature extraction process and image processing tasks [33]. CAE uses a convolution operator for encoding the input features and replicating them in the output with a minimal amount of reconstructed errors. CAE consists of output layer m feature maps and m convolution kernels. The input mapping feature is generated from the input layer while n corresponds to the number of input channels. The hidden depiction of CAE of the $k^{th}$ feature map in the encoder is described using Equation (12), where σ denotes the activation function and ∗ indicates the 2D convolution. In the decoder, the reconstruction is described using a subsequent equation, where H shows the hidden feature maps and c denotes the bias as per the input channel [34].

(12) $$h^{k}=\sigma \left(x\;*\;W^{k}+b^{k}\right),\;\;$$

(13) $$y=\sigma \left(\sum_{k\epsilon H}h^{k}\;*\;{\tilde{W}}^{k}+c\right)\;\;$$

### 3.3. Stage III: Parameter Tuning Using the TEO Method

Ultimately, the TEO has been implemented in the current study for fine-tuning the parameters, compared to the CNN-AE architecture. The target of hyperparameter selection is critical for fine-tuning the configuration of the CNN-AE technique. Optimum hyperparameters considerably impact the effectiveness of the model, while they also affect the model’s capability for effectually taking complex features and generalizing them. By implementing the TEO technique, the research goal is to proficiently direct the hyperparameter space and enhance the capabilities of CNN-AEs in the context of CCP within the telecom industry. The TEO method is inspired from the unique ability to represent the principles of thermal equilibrium in physical systems, thus enabling a robust analysis of the hyperparameter space. The TEO system provides different benefits in the optimization process, mainly in hyperparameter tuning for the DL models. Inspired from the principles of thermal equilibrium, the TEO technique strikes an active balance between the exploration and exploitation phases. Thus, it can navigate complex solution spaces, mimic physical methods, provide greater convergence and solution quality, and can be combined with local and global search approaches. The versatility and efficiency of the TEO method make it a favorable choice for fine-tuning the hyperparameters in architectures, namely CNN-AE. Further, it is also applicable in case of CCP in the telecom industry, where it yields an enriched performance and can accomplish optimum configurations.

According to the Newton’s law of cooling, TEO is a novel optimization technique, which describes that the rate of heat loss for an object is directly proportionate to the temperature difference between the object and its surrounding environments at a certain point [35]. In the current research work, some search agents are represented as reference, while some as recognized nodes (cooling objects). Unrecognized NLOS nodes or nodes, on the other hand, are represented as environment. The heat exchange between the environment and the cooling objects is mathematically modelled as follows:

(14) $$T_{i}^{c-env}=\left(1-\left(cv_{1}+cv_{2}\;*\;\left(1-N_{CI}\right)\;*\;rnd\right)\right)\;*\;T_{i}^{p-env}$$

(15) $$N_{CI}=\frac{C_{IN}}{{Max}_{Iter}}\;\;\;$$

$T_{i}^{p-env}$ and $T_{i}^{x-env}$ represent the earlier and the modified temperatures of the environment’s objects, respectively, with $cv_{1}$ and $cv_{2}$ being considered as the variables used for controlling the prediction or localization operations, correspondingly [36]. Furthermore, $C_{IN}$ and MaxIter refer to the existing and the maximum iteration counts. In addition to this, the initial phase of the TEO optimization technique updates the temperature of the objects and their surrounding environments as given below.

(16) $$T_{i}^{new-env}=T_{i}^{x-env}+\left(T_{i}^{old-env}-T_{i}^{x-env}\right)\;*\;e^{-\beta N_{CI}}$$

(17) $$\beta =\frac{CosineN_{CI}(Obi)}{CosineN_{CI}(Worst\_Obj)}\;\;$$

Now, the rnd value is compared to the predefined prevention threshold that has been implemented earlier for randomly selecting a single dimension of the $i^{th}$ searching agent to restore its value based on Equation (18):

(18) $$T_{i,j}=T_{i,Min}+rnd\;*\;\left(T_{j,Max}-T_{j,Min}\right)$$

In Equation (18), $T_{j}$ represents the $j^{th}$ variable of the $i^{th}$ searching agent, with T, Min and T,Max correspondingly indicating the lower and upper thresholds of the $j^{th}$ variable [37]. Fitness selection has been an essential component in the TEO methodology. An encoder solution is applied to estimate the outcome of the solution candidate. Therefore, the accuracy value is the foremost form applied for designing the FF.

(19) $$Fitness=max\left(\frac{TP}{TP+FP}\right)\;$$

Here, the true and false positive values are denoted by TP and FP, respectively. 

## 4. Results and Discussion

The developed method was validated using the Python 3.8.5 tool on a PC configured with i5-8600k, GeForce 1050Ti 4 GB, 16 GB RAM, 250 GB SSD, and 1 TB HDD specifications. Diverse Python Packages were implemented, namely opencv-python, numpy, matplotlib, tensorflow (GPU-CUDA Enabled), keras, pickle, sklearn, and pillow. The CCP performance of the AOAFS-HDLCP technique was investigated using the customer churn prediction: Telecom Churn Dataset [38], including 3,333 data instances with 21 attributes as described in Table 1. The dataset was downloaded from the Kaggle repository.

The set of measures, used for examining the classification outcomes, are accuracy ($accu_{y}$), precision ($prec_{n}$), recall ($reca_{l}$), and F-score ($F_{score}$).

(20) $$Prec_{n}=\frac{TP}{TP+FP}$$

Precision is used to measure the proportion of the predicted positive instances out of each instance that is predicted as positive.

(21) $$Reca_{l}=\frac{TP}{TP+FN}$$

Recall is used to measure the proportion of the positive samples classified.

(22) $$Accu_{y}=\frac{TP+TN}{TP+TN+FP+FN}$$

Accuracy is used to measure the proportion of the classified samples (positive and negative) against the overall samples classified.

(23) $$F_{score}=\frac{2TP}{2TP+FP+FN}$$

F-score combines the harmonic mean of $prec_{n}$ and $reca_{l}$.

**Figure 3:**
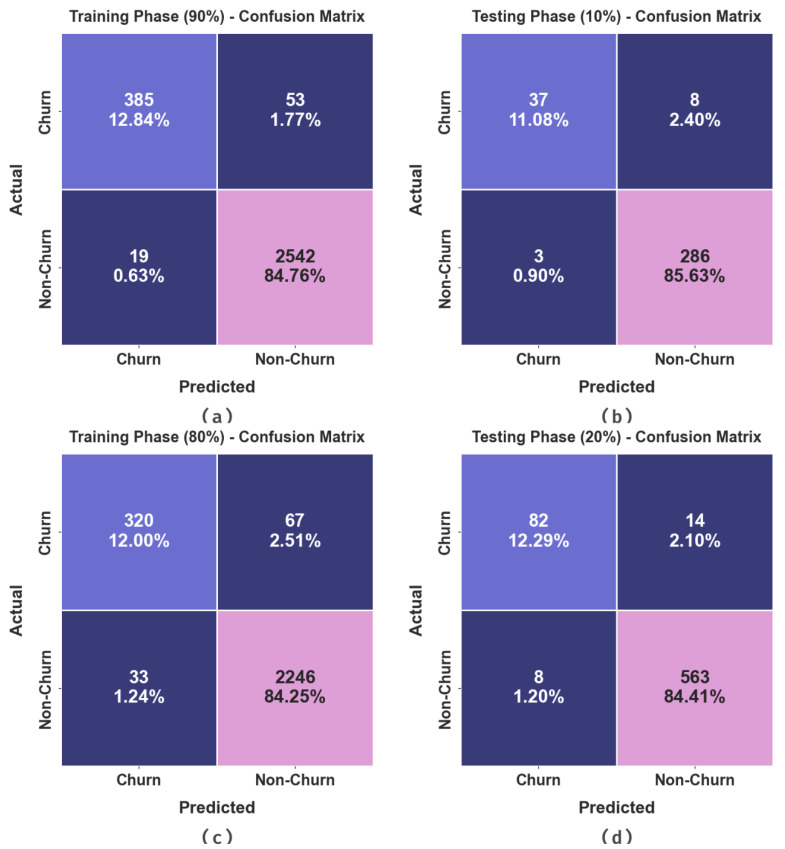
Confusion matrices of (**a**,**b**) 90:10 of TR set (TRS)/TS set (TSS) and (**c**,**d**) 80:20 of TRS/TSS.

The confusion matrices generated by the AOAFS-HDLCP method on 90:10 and 80:20 of the TRS/TSS datasets are demonstrated in Figure 3. The outcomes portray the effectual recognition of the proposed model in terms of churn and non-churn samples on all the class labels.

The CCP outcomes of the AOAFS-HDLCP method under 90:10 and 80:20 of the TRS/TSS datasets are shown in Table 2. The simulation values demonstrate that the AOAFS-HDLCP method categorized the churn and non-churn samples effectively. With 90% TRS, the AOAFS-HDLCP model provided an average $accu_{y}$ of 93.58%, $prec_{n}$ of 96.63%, $reca_{l}$ of 93.58%, $F_{score}$ of 95.03%, and an $AUC_{score}$ of 93.58%. In addition, with 10% TSS, the AOAFS-HDLCP technique offered an average $accu_{y}$ of 90.59%, $prec_{n}$ of 94.89%, $reca_{l}$ of 90.59%, $F_{score}$ of 92.59%, and an $AUC_{score}$ of 90.59%. Also, with 80% TRS, the AOAFS-HDLCP model yielded an average $accu_{y}$ of 90.62%, $prec_{n}$ of 93.88%, $reca_{l}$ of 90.62%, $F_{score}$ of 92.15%, and an $AUC_{score}$ of 90.62%. At last, with 20% TSS, the AOAFS-HDLCP method accomplished an average $accu_{y}$ of 92.01%, $prec_{n}$ of 94.34%, $reca_{l}$ of 92.01%, $F_{score}$ of 93.13%, and an $AUC_{score}$ of 92.01%.

**Figure 4:**
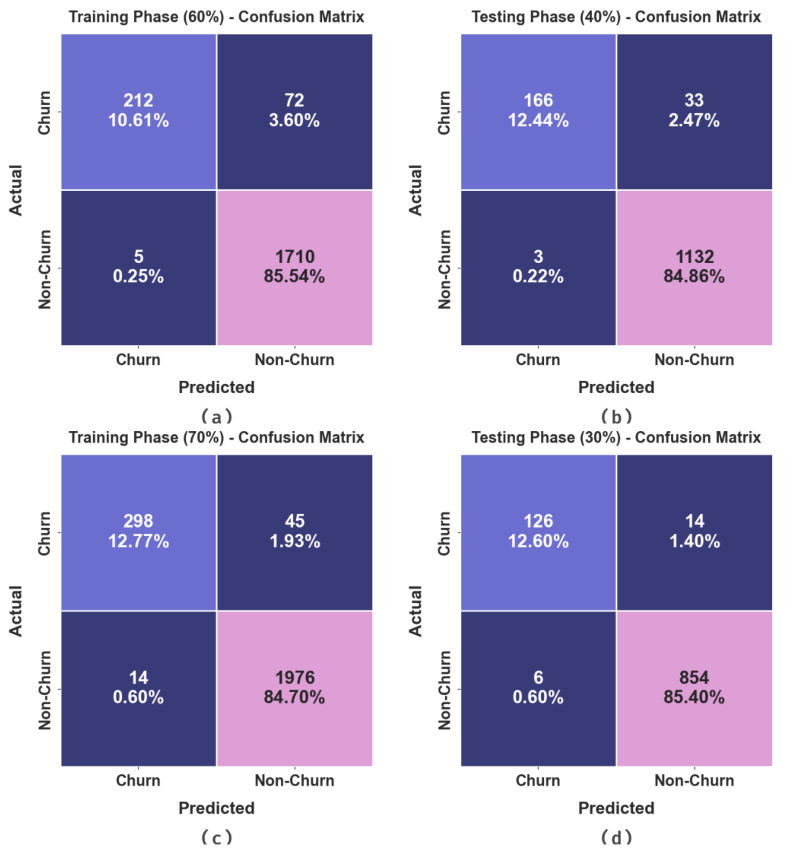
Confusion matrices of (**a**,**b**) 60:40 of TRS/TSS and (**c**,**d**) 70:30 of TRS/TSS.

The confusion matrices generated by the AOAFS-HDLCP system on 60:40 and 70:30 TRS/TSS datasets are illustrated in Figure 4. The outcomes indicate the effectual prediction of the proposed model in terms of churn and non-churn samples under all the classes.

The CCP outcomes of the AOAFS-HDLCP system at 60:40 and 70:30 TRS/TSS datasets are shown in Table 3. The achieved outcomes indicate that the proposed AOAFS-HDLCP technique categorized the churn and non-churn samples in an effective manner. With 60% TRS, the AOAFS-HDLCP method provided an average $accu_{y}$ of 87.18%, $prec_{n}$ of 96.83%, $reca_{l}$ of 87.18%, $F_{score}$ of 91.21%, and an $AUC_{score}$ of 87.18%. In addition, with 40% TSS, the AOAFS-HDLCP method yielded an average $accu_{y}$ of 91.58%, $prec_{n}$ of 97.70%, $reca_{l}$ of 91.58%, $F_{score}$ of 94.33%, and an $AUC_{score}$ of 91.58%. Also, with 70% TRS, the AOAFS-HDLCP method produced an average $accu_{y}$ of 93.09%, $prec_{n}$ of 96.64%, $reca_{l}$ of 93.09%, $F_{score}$ of 94.76%, and an $AUC_{score}$ of 93.08%. At last, with 30% TSS, the AOAFS-HDLCP method accomplished an average $accu_{y}$ of 94.65%, $prec_{n}$ of 96.92%, $reca_{l}$ of 94.65%, $F_{score}$ of 95.74%, and an $AUC_{score}$ of 94.65%.

**Figure 5:**
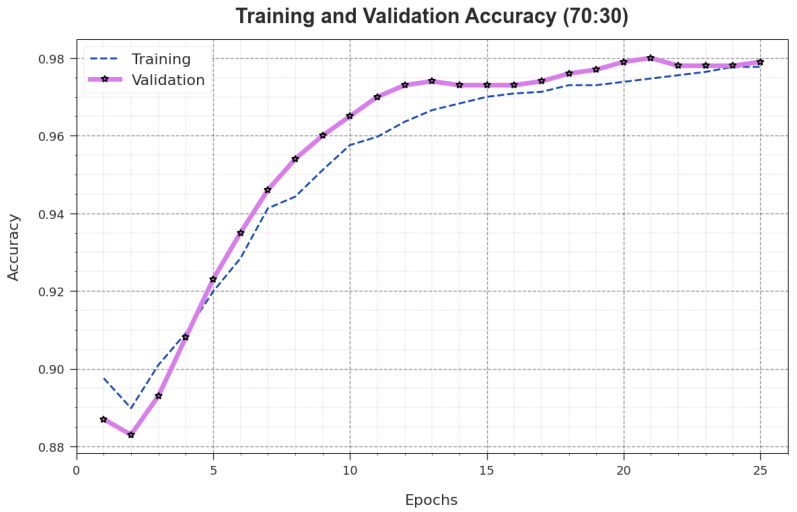
$Accu_{y}$ curve of AOAFS-HDLCP method on 70:30 of TRS/TSS.

Both $TR\_accu_{y}$ and $VL\_accu_{y}$ outcomes of the AOAFS-HDLCP methodology for 70:30 TRS/TSS dataset are illustrated in Figure 5. The $TL\_accu_{y}$ is evaluated by estimating the AOAFS-HDLCP system on the TR data, while $VL\_accu_{y}$ is determined by the assessment of the proposed method using test data. The simulation values show that both $TR\_accu_{y}$ and $VL\_accu_{y}$ values increase with the maximum number of epochs. Hereafter, the effectiveness of the AOAFS-HDLCP method increases on the TR and TS data with an increase in the number of epochs. 

**Figure 6:**
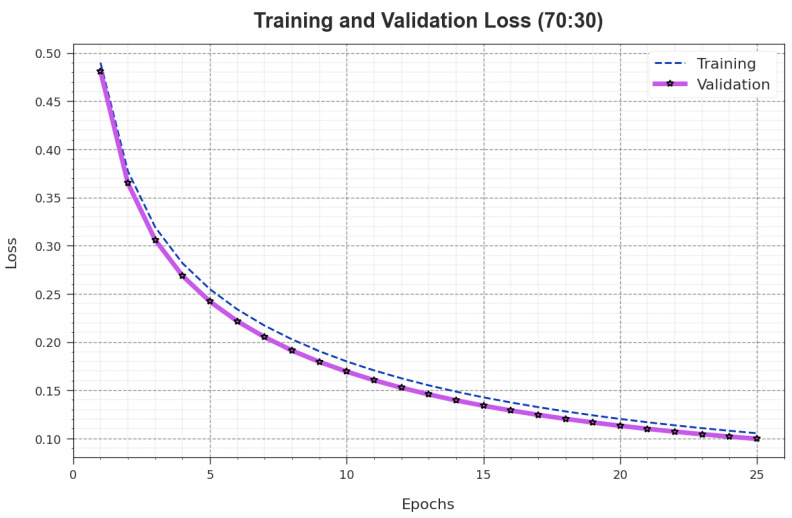
Loss curve of the AOAFS-HDLCP model under 70:30 of TRS/TSS.

The TR_loss and VR_loss outcomes of the AOAFS-HDLCP model under 70:30 of the TRS/TSS are shown in Figure 6. The TR_loss represents the error between the prediction performance and original values at the TR dataset. The VR_loss denotes the performance evaluation of the AOAFS-HDLCP method on the validation dataset. The simulation value demonstrates that both TR_loss and VR_loss tend to reduce with an increase in the number of epochs. This provides the superior outcome of the AOAFS-HDLCP algorithm and its ability to produce accurate classification. The minimized TR_loss and VR_loss values reveal the high efficiency of the AOAFS-HDLCP system in capturing patterns and correlations.

**Figure 7:**
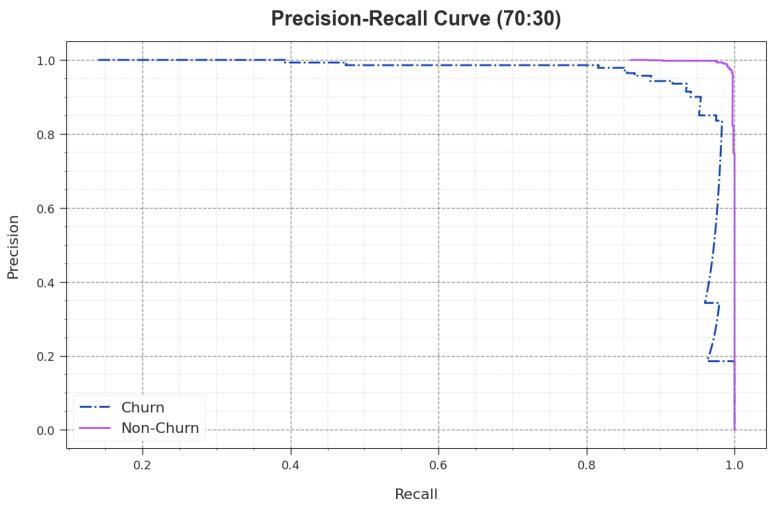
PR analysis of the AOAFS-HDLCP methodology under 70:30 of TRS/TSS.

A wide range of PR analysis was conducted upon the AOAFS-HDLCP model upon the 70:30 TRS/TSS dataset and the results are shown in Figure 7. The simulation values infer that the AOAFS-HDLCP approach produced the maximum PR values. Additionally, the AOAFS-HDLCP technique attained the maximum PR performance in all the classes.

**Figure 8:**
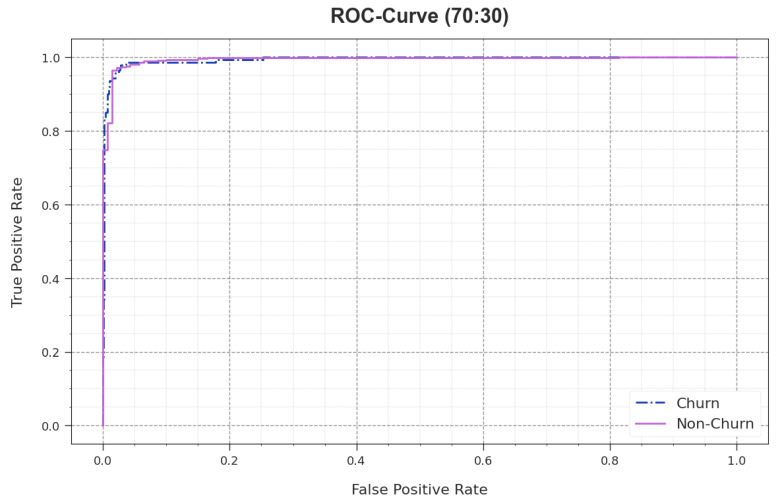
ROC of AOAFS-HDLCP model under 70:30 of TRS/TSS.

In Figure 8, the ROC analysis curve achieved by the AOAFS-HDLCP algorithm for 70:30 TRS/TSS dataset is shown. This figure indicates that the AOAFS-HDLCP system achieved an improvement in the ROC values. The outcomes provide valuable insights about the tradeoffs between the rate of TPR and FPR. It provides the predictive outcomes of the presented technique on the classification of different classes.

Table 4 shows the results of the comparison analysis conducted between the proposed AOAFS-HDLCP method and the existing methods [20,39,40]. The experimental values infer that the DR and LR models exhibited poor results, whereas the SVM, SGD, and RMSProp approaches achieved slightly increased performance.

## 5. Conclusions

In the current study, the AOAFS-HDLCP technique has been introduced for churn prediction in the telecom industry. The objective of the presented method is to accomplish churn prediction so as to increase the customer retention process in the telecom industry. In the presented technique, the AOAFS approach, CNN-AE classification, and TEO-based hyperparameter tuning have been developed. In the current research work, the AOAFS is designed to choose an optimal set of features. The CNN-AE model has been involved in churn prediction process. The TEO technique has been applied to the hyperparameter tuning process to optimize the outcomes of the CNN-AE system. A widespread experimental analysis was conducted to illustrate the superior performance of the AOAFS-HDLCP approach. The achieved findings portray the significant performance of the AOAFS-HDLCP method over other techniques, with an improved accuracy of 94.65%. In the future, studies can focus on handling outlier removal and class imbalance data handling problems.

## Figures and Tables

**Table 1:** Details of the database.

Class	No. of Samples
Churn	483
Non-Churn	2850
Total Samples	3333

**Table 2:** CCP outcomes of the AOAFS-HDLCP method on 90:10 and 80:20 of TRS/TSS datasets.

Class	$Accu_{y}$	$Prec_{n}$	$Reca_{l}$	$F_{score}$	$AUC_{score}$
Training Phase (90%)					
Churn	87.90	95.30	87.90	91.45	93.58
Non-Churn	99.26	97.96	99.26	98.60	93.58
Average	93.58	96.63	93.58	95.03	93.58
Testing Phase (10%)					
Churn	82.22	92.50	82.22	87.06	90.59
Non-Churn	98.96	97.28	98.96	98.11	90.59
Average	90.59	94.89	90.59	92.59	90.59
Training Phase (80%)					
Churn	82.69	90.65	82.69	86.49	90.62
Non-Churn	98.55	97.10	98.55	97.82	90.62
Average	90.62	93.88	90.62	92.15	90.62
Testing Phase (20%)					
Churn	85.42	91.11	85.42	88.17	92.01
Non-Churn	98.60	97.57	98.60	98.08	92.01
Average	92.01	94.34	92.01	93.13	92.01

**Table 3:** CCP outcomes of the AOAFS-HDLCP method on 60:40 and 70:30 of TRS/TSS datasets.

Class	$Accu_{y}$	$Prec_{n}$	$Reca_{l}$	$F_{score}$	$AUC_{score}$
Training Phase (60%)					
Churn	74.65	97.70	74.65	84.63	87.18
Non-Churn	99.71	95.96	99.71	97.80	87.18
Average	87.18	96.83	87.18	91.21	87.18
Testing Phase (40%)					
Churn	83.42	98.22	83.42	90.22	91.58
Non-Churn	99.74	97.17	99.74	98.43	91.58
Average	91.58	97.70	91.58	94.33	91.58
Training Phase (70%)					
Churn	86.88	95.51	86.88	90.99	93.09
Non-Churn	99.30	97.77	99.30	98.53	93.09
Average	93.09	96.64	93.09	94.76	93.09
Testing Phase (30%)					
Churn	90.00	95.45	90.00	92.65	94.65
Non-Churn	99.30	98.39	99.30	98.84	94.65
Average	94.65	96.92	94.65	95.74	94.65

**Table 4:** Comparison analysis outcomes of the AOAFS-HDLCP technique with other approaches [20,39,40].

Methods	$Accu_{y}$	$Prec_{n}$	$Reca_{l}$	$F_{score}$	$AUC_{score}$
AOAFS-HDLCP	94.65	96.92	94.65	95.74	94.65
AIJOA-CPDE	91.28	95.52	91.29	94.08	91.29
Logistic Regression	80.53	79.31	80.44	79.05	82.18
Decision Tree	76.67	56.78	75.68	64.97	78.25
ISMOTE-OWELM	90.48	91.65	89.39	89.64	89.85
SVM Model	84.29	84.54	83.99	85.59	83.98
SGD Model	84.41	86.10	85.81	84.32	84.80
RMSProp Model	87.35	85.18	85.19	85.07	86.27

Along with that, the AIJOA-CPDE approach illustrated reasonable outcomes with an $accu_{y}$ of 91.28%, $prec_{n}$ of 95.52%, $reca_{l}$ of 91.29%, $F_{score}$ of 94.08%, and an $AUC_{score}$ of 91.29%. However, the AOAFS-HDLCP technique gained the maximum performance with an $accu_{y}$ of 94.65%, $prec_{n}$ of 96.92%, $reca_{l}$ of 94.65%, $F_{score}$ of 95.74%, and an $AUC_{score}$ of 94.65%. Therefore, the AOAFS-HDLCP technique can be applied for accurate CCP process.

## Data Availability

Data sharing does not apply to this article as no datasets were generated during the current study.
